# Endoscopic annular chondroperichondrial tympanoplasty, technical description

**DOI:** 10.1007/s00405-025-09238-7

**Published:** 2025-01-25

**Authors:** M. Ihsan Gülmez, Şemsettin Okuyucu

**Affiliations:** Hatay Mustafa Kemal University Otorhinolaryngology Department, Hatay, Turkey

**Keywords:** Endoscopic ear surgery, Tympanoplasty, Cartilage graft, Fascia graft

## Abstract

**Purpose:**

Tympanoplasty is a surgical procedure performed to cure middle ear infections and restore normal middle ear function. It is one of the most common procedures in otological surgery. Since Wullstein described tympanoplasty, the microscope has been a widely used surgical tool in otological surgery. Transcanal endoscopic ear surgery (TEES) has come to the forefront as an alternative option due to the various limitations of microscopic surgery and the development of otological surgical techniques. When reviewing the types and tecniques of grafts used in tympanoplasty in the current literature, it is clear that the ideal graft material and tecnique to meet all needs has not yet been found. The objective of this study is to describe a novel graft technique, a modification of the cartilage-perichondrium graft obtained from tragal cartilage, which is believed to represent an optimal solution for the desired graft technique at the highest level.

**Material&Method:**

The study included 40 patients who underwent endoscopic annular chondroperichondrial tympanoplasty at Hatay Mustafa Kemal University Hospital between 2015 and 2022. All patients underwent clinical otological examination and pure tone audiometry before and after surgery. Hearing results were expressed as pure tone mean air-bone gap (ABG) at 4 frequencies (500,1000,2000,4000 Hz). For all operations, the primary surgeon was the second author. Patients with inadequate follow-up, revision surgery, inflammatory middle ear mucosa, perforation etiology other than chronic otitis media were not included in the study.

**Results:**

No complications were observed in the patients included in the study. Accordingly, the graft was intact at the end of the 6th month in 38 of the 40 patients operated on. The graft success rate was 95%. The mean preoperative PTA-ABG was 22.1. The mean post-operative PTA-ABG was 5.7. Mean closed PTA-ABG was 16.4.

**Conclusion:**

In parallel with the development of otological surgical technologies, endoscopy is becoming increasingly important in tympanoplasty. We believe that our technique, endoscopic annular chondroperichondrial tympanoplasty, will be an important step in the search for the ideal grafting technique.

## Introduction

Tympanoplasty is a surgical procedure performed to cure middle ear infections and restore normal middle ear function [1]. It is one of the most common procedures in otological surgery. The first attempt to close a perforated eardrum dates back many years. Banzer used a deer claw tube covered with pig bladder in 1644 [2, 3]. Although various definitions have been made since then, following the development of surgical microscopes and instruments, Wullstein defined and classified tympanoplasty in the 1950s [4]. According to this classification, type I consists of a simple TM perforation with a normal ossicular chain, while type V describes stapes footplate fixation with lateral semicircular canal fenestration [4]. These advances in microscopic technology have allowed the development of many otological microsurgical techniques, including modern tympanoplasty.

Transcanal endoscopic ear surgery (TEES) has come to the forefront as an alternative option due to the various limitations of microscopic surgery and the development of otological surgical techniques. Although TEES was first reported in 1967 by Dr Mer et al., it became popular after the 1990s [5]. It is being used more and more every day, mainly because it overcomes the difficulty of a straight line of sight in microscopic surgery. With this feature, TEES provides high-resolution and superior visualisation of the tympanic membrane without the need for additional intervention in patients with narrow and tortuous external auditory canal [6]. In addition, its favourable features such as shorter hospital stay [6, 7], shorter operation time [8–10], better cosmetic results [2, 7, 11] and less postoperative pain [2, 6, 8, 12] compared to microscopic surgery have been reported in various studies in the literature.

Since Wullstein’s description of tympanoplasty, a variety of graft materials have been used to repair the tympanic membrane. These include temporal muscle fascia, perichondrium, fat, cartilage, skin, dura mater and vein [13]. Temporal muscle fascia and cartilage grafts are the most commonly used graft materials today [14]. Temporal muscle fascia has been preferred for many years due to its semi-permeability, thin structure, easy accessibility, easy availability and availability in large quantities [14]. However, it has a structure that is not resistant to pressure changes due to its low elasticity [14]. Therefore, unsuccessful results can be achieved in cases such as Eustachian tube dysfunction, adhesive otitis media, retraction pockets and large or complete perforation when using temporal muscle fascia [13]. More rigid materials that are more resistant to infection, resorption and retraction are required in these cases [13]. Cartilage transplants, with their high elasticity, are flexible and more resistant to pressure changes and may be a more appropriate choice in the above pathologies [14].

When reviewing the types of grafts used in tympanoplasty in the current literature, it is clear that the ideal graft material and technique to meet all needs has not yet been found. Each graft material and technique has its own positive and negative characteristics. The objective of this study is to describe a novel graft technique, a modification of the cartilage-perichondrium graft obtained from tragal cartilage, which is believed to represent an optimal solution for the desired graft technique at the highest level. We believe that the endoscopic annular chondroperichondrial tympanoplasty technique, which has maximum graft integration, achieves optimal hearing, has sufficient physical resistance, does not complicate middle ear follow-up due to its semi-permeable nature, is easy to shape and can be easily applied endoscopically or microscopically, will contribute to the literature.

## Material & method

The study included 40 patients who underwent endoscopic annular chondroperichondrial tympanoplasty at Hatay Mustafa Kemal University Hospital between 2015 and 2022. The aetiology of perforation in all patients was chronic otitis media for at least 1 year prior to surgery. All patients had dry and non-infected middle ear mucosa for at least 1 month prior to surgery. Perforations measuring 3 × 3 mm and subtotal range, as well as perforations involving the manibrium mallei in terms of localisation, were deemed to be suitable for the technique. The perforations were categorised as above or below 50% based on size criteria. Perforations measuring less than 3 × 3 mm or larger than 3 × 3 mm, which could be treated with a less invasive technique that would not necessitate malleus desepithelialisation, were treated with the optimal technique, which included procedures such as fat myringoplasty and butterfly cartilage tympanoplasty, amongst others, and these patients excluded from the study. The patients included in the study were selected based on the criteria that the endoscopic annular chondroperichondrial tympanoplasty technique was deemed to be the most optimal treatment option, given the specific characteristics of their pathology. All patients underwent clinical otological examination and pure tone audiometry before and after surgery. Postoperative follow-up was scheduled at 10 days, 1 month, 6 months, 1 year, and annually thereafter. Hearing results were expressed as pure tone mean air-bone gap (ABG) at 4 frequencies (500,1000,2000,4000 Hz). For all operations, the primary surgeon was the second author. Patients with inadequate follow-up, revision surgery, inflammatory middle ear mucosa, perforation etiology other than chronic otitis media were not included in the study. The study was approved by the Hatay Mustafa Kemal University Ethics Committee, with the decision numbered 2024/09/31.

### Surgical technique

**Fig. 1:**
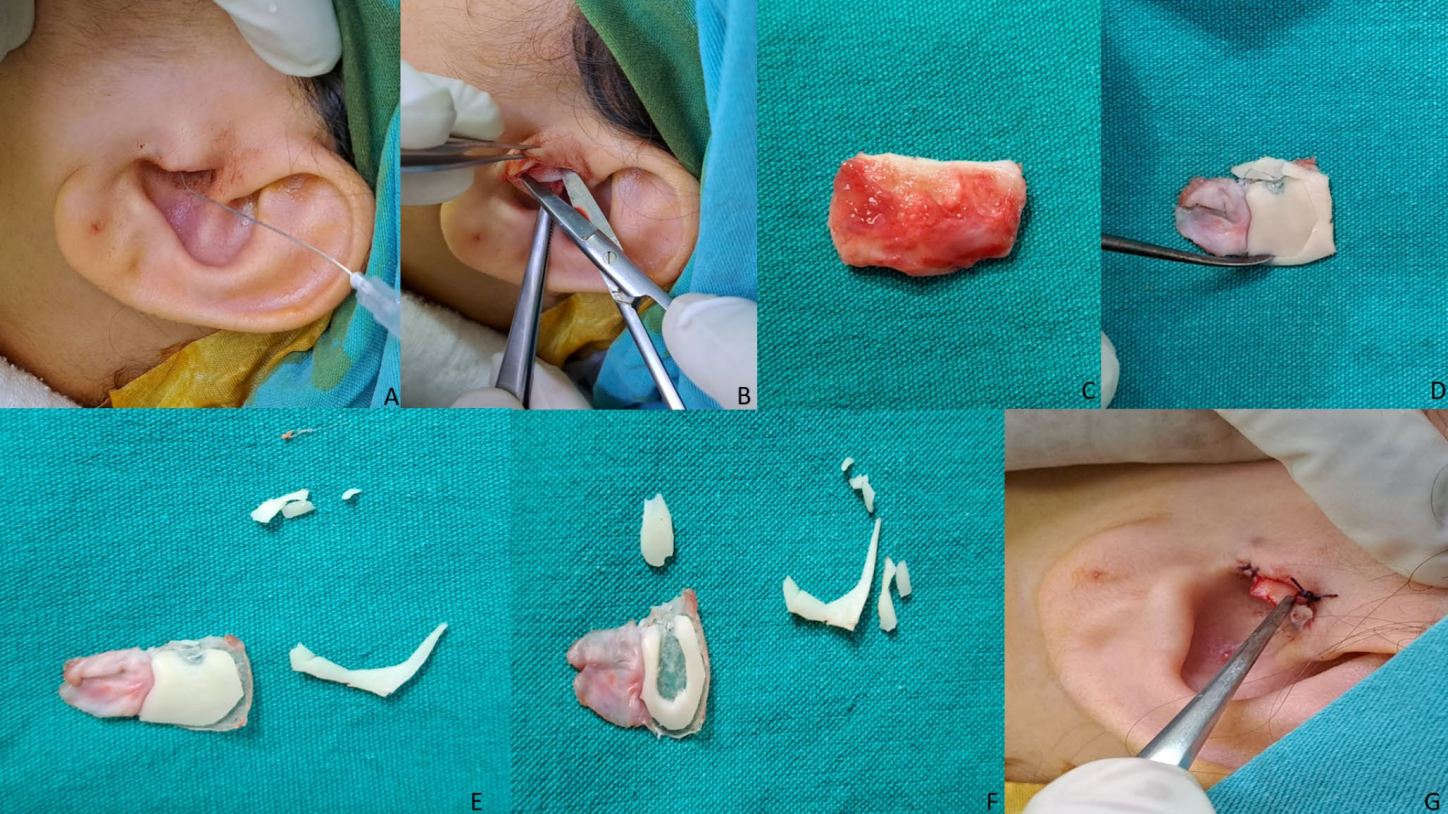
**A**: Injection of local anaesthetic into the tragus, **B**: An incision is made on the inner surface of the tragus and the tragal cartilage island graft is harvested, **C**: The tragal cartilage island graft, which has not yet been shaped, is to be used in surgery. **D**: The perichondrium on the side of the graft facing the middle ear was peeled off and opened 180 degrees. **E**: A ring-shaped excision of approximately 1 mm from the periphery of the cartilage section was then planned to create a cartilage graft with a free perichondrial edge **F**: From the centre of the remaining cartilage-perichondrium graft, only cartilage tissue was removed in a circular fashion, leaving approximately 2–3 mm of ring-shaped cartilage. Subsequently, a slot was created in the region where the manibrium of the malleus would be positioned in alignment. **G**: The remaining cartilage tissues were repositioned within the original anatomical location

All surgery was performed under general anaesthesia. Total intravenous anaesthesia was preferred and a mean blood pressure of 60 mm/Hg and a heart rate of 60 bpm were targeted. A 3 mm straight telescope tip (Karl Storz 7220 AA) was used for all operations. A mixture of 20 mg/ml lidocaine hydrochloride + 0.0125 mg/ml adrenaline was used as local anaesthetic. At the beginning of the operation, local anaesthetic was injected into the tragus and the external auditory canal (Fig. 1A). The hairs in the external auditory canal were removed if necessary. The cartilage graft was harvested from the tragus cartilage. An incision was made from the posterior edge of the tragus, leaving a 2 mm border cartilage for cosmetic reasons (Fig. 1B). The remaining tragal cartilage island graft was excised and brought to the table for shaping (Fig. 1C). Easily accessible surgical instruments such as a size 11 scalpel, size 15 scalpel, duckbill and flat-tipped pick were used to shape the cartilage. The perichondrium on the side of the cartilage graft facing the middle ear was completely peeled off and either completely removed or left on the graft by opening it 180 degrees to be placed in the external auditory canal, as required (Fig. 1D). The cartilaginous part of the graft to be formed was removed by cutting a 1 mm ring of cartilage from the periphery. In this way, approximately 1 mm of free perichondrium was created and better graft integration was aimed for (Fig. 1E). Then, from the centre of the remaining cartilage-perichondrium graft, only cartilage tissue was removed in a circular fashion, leaving approximately 2–3 mm of ring-shaped cartilage. This left a chondroperichondrium graft with approximately 2–3 mm of ring cartilage. Considering the under-over laying of the graft, the cartilage was also removed in the part where it would correspond to the thickness of the manibrium mallei and a slot was created for the manibrium mallei (Fig. 1F). The remaining cartilage tissues were repositioned within the original anatomical location (Fig. 1G). The graft was prepared for use.

**Fig. 2:**
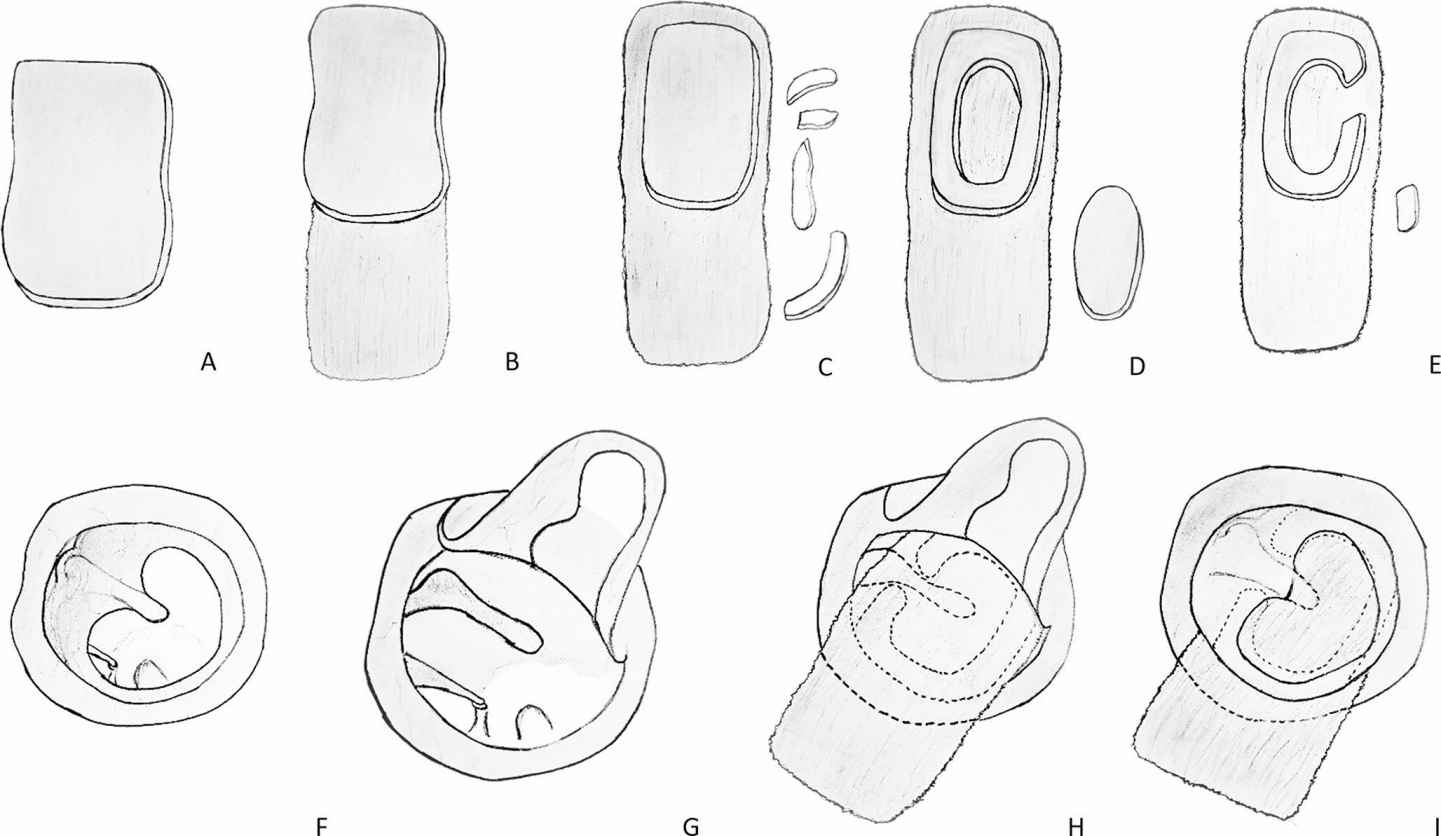
**A**: The tragal cartilage tissue is not yet formed **B**: The cartilage graft is opened 180 degrees by peeling the perichondrium on the side facing the middle ear **C**: A ring-shaped excision of approximately 1 mm from the periphery of the cartilage section was then planned to create a cartilage graft with a free perichondrial edge **D**: From the centre of the remaining cartilage-perichondrium graft, only cartilage tissue was removed in a circular fashion, leaving approximately 2–3 mm of ring-shaped cartilage **E**: A slot was created in the region where the manibrium of the malleus would be positioned in alignment **F**: The appearance of the tympanic membrane with a central perforation following aviation. **G**: View of the middle ear following the elevation of the tympanomeatal flap **H**: Under-over placement of prepared annular cartilage chondroperichondrial graft **I**: The final image of the operation depicts the tympanomeatal flap returned to its original position

After aviation of the perforation edges (Fig. 2F), an incision was made in the external auditory canal infiltrated with local anaesthetic at 6–12 o’clock and the tympanomeatal flap was elevated. The flap was elevated to the bony annulus and the middle ear was entered superiorly through the Rivinius notch. The flap was gently separated from the malleus and adhered to the anterior wall (Fig. 2G). After the planned middle ear surgery, if any, was performed, the graft was supported with gelfoam. The graft was placed using the under-over technique so that the malleus fit into the notch created (Fig. 2H). After ensuring that the graft was balanced in all quadrants (Fig. 2I), the Gelfoam support was placed in the external auditory canal. The procedure was completed by placing a sponge dressing over the auricle.

## Results

Demographic information and examination findings of the patients are shown in Table 1. Five of the patients were under 18 years old and 35 were adults. The age of the patients ranged from 12 to 68 years, with a mean age of 39.15 years. A total of 14 male and 26 female patients were included in the study. In 23 patients, the affected ear was retained, while in 17 patients, the right ear was retained. The perforation size was less than 50% in 21 patients and greater than 50% in 19 patients. No complications were observed in the patients included in the study. Adequate and complete integration of the graft at the end of the follow-up period of at least 6 months after surgery was considered a successful operation. Accordingly, the graft was intact at the end of the 6th month in 38 of the 40 patients operated on. The graft success rate was 95% (Table 2). In 2 patients in whom the graft was not intact, patency was achieved with minor revision procedures during the follow-up period. The mean preoperative PTA-ABG was 22.1. The mean post-operative PTA-ABG was 5.7. Mean closed PTA-ABG was 16.4 (Table 3).

**Table 1:** Patients’ demographic information and examination findings

Age, (years)	
Mean Range	39,15 12–68
Gender, n (%)	
Male Female	14 ( 35) 26 ( 65)
Affected side, n (%)	
Left Right	23 ( 57,5) 17 ( 42,5)
Perforation size	
<%50 >%50	21 (52,5) 19 (47,5)

**Table 2:** Greft closure rate

*Greft closure*	*Perforation size*	
	<%50	>%50
Succesfull	20	18
Unsuccesfull	1	1

## Discussion

Since Wullstein described tympanoplasty, the microscope has been a widely used surgical tool in otological surgery. The combination of precise three-dimensional vision with ideal magnification has greatly improved otological surgery, enabling surgeons to perform effective and safe surgery. However, the flat viewing angle of the microscope and the unique anatomy of the external auditory canal have emerged as challenges in microscopic surgery [15]. TEES, with its wide-angle optics and close proximity of the light source to the surgical field, creates an ideal surgical environment without the need for additional procedures, even in patients with a narrow and curved external auditory canal [16].

There are several studies in the literature comparing endoscopic and microscopic surgery and analysing their advantages and disadvantages. The main advantages of the microscope, which has been used in ear surgery for many years, are that it provides three-dimensional vision and allows the active use of both hands in the surgical field [8, 9]. The most important feature of endoscopy is the wide angle and high quality image of the surgical field [2, 16]. It allows access to the surgical field without the need for an additional procedure through the external auditory canal, thus eliminating the need for incisions and external auditory canal procedures in microscopic surgery. Because of its minimally invasive nature, the endoscope is superior to the microscope in terms of pain, operating time, cosmesis and return to work [2, 6–12]. Endoscopic surgery has a shorter operating time due to the image quality of the surgical field and the absence of invasive procedures such as incision and closure [8–10]. Again, due to its minimally invasive nature, the hospital stay is shorter and the cost of surgery is reduced [2, 6, 7]. In their 2017 study, Drs. Kuo and Wu reported the cost of endoscopic tympanoplasty to be 645 euros and the cost of microscopic tympanoplasty to be 1170 euros [17]. In their meta-analysis, Dr Crotty et al. reported that the graft success rates of microscopic and endoscopic surgery were similar and around 90%, with endoscopic surgery having a better postoperative ABG closure rate, better pain score, shorter operative time and shorter hospital stay [6]. In a meta-analysis comparing endoscopic and microscopic surgery in paediatric patients, Dr Han et al. reported similar graft success rates, similar recurrence rates after cholesteatoma surgery and better control of residual cholesteatoma with endoscopic surgery [10]. Current disadvantages of endoscopic surgery include the need for one-handed surgery, frequent lens fogging, loss of binocular vision and depth perception, potential risk of thermal injury, rapid bleeding, and difficulty working with additional surgical instruments such as a tourniquet [2, 8, 10, 16].

Reorganisation of the middle ear conduction mechanism requires an intact and secure connection between an intact tympanic membrane and the inner ear fluids [1]. A well ventilated middle ear cavity with a healthy mucosa should be covered by an intact tympanic membrane. Impedance is generally defined as an obstacle or resistance to movement [18]. Acoustic impedance can be defined as the resistance of the system to the passage of energy during the passage of sound energy and is the ratio of the sound pressure to the volume velocity produced by the sound pressure [18]. One of the most important functions of the middle ear is impedance compensation. Acoustic impedance has three important components: stiffness, resistance and mass effect [18]. Therefore, as the stiffness, resistance and mass effect of the tympanic membrane increase, the volume velocity generated by the acoustic stimulus will decrease. This will have a negative effect on the hearing performance of the middle ear.

Given these parameters of auditory physiology, temporal muscle fascia graft was used as the graft that would best fulfil the function of the ideal tympanic membrane. It is the most commonly used graft material with a 93–97% success rate in primary tympanoplasty [13]. The main reasons for this preference are its semi-permeability, thin structure, easy accessibility, easy availability and availability in large quantities [14]. However, due to its low elasticity, it has a structure that is not resistant to pressure changes and therefore unsuccessful results may be obtained in cases such as Eustachian tube dysfunction, adhesive otitis media, retraction pockets and large or total perforation [13, 14]. The cartilage graft is flexible due to its high elasticity and is more resistant to pressure changes [14]. In addition, the fact that it is supplied by diffusion facilitates patch acceptance [14, 16]. Despite these favourable characteristics, it is thought that the excessive thickness, stiffness and mass of the cartilage graft may adversely affect graft integration and hearing physiology [19].

Since the beginning of modern otological surgery in the 1950s, microscopic overlay tympanoplasty has been the standard surgical approach [20]. In the overlay technique, the graft is placed lateral to the fibrous layer of the tympanic membrane remnant and medial to the malleus [1]. It requires complete peeling of the squamous cell epithelium on the lateral surface of the tympanic membrane remnant to avoid iatrogenic cholesteatoma formation, canalplasty for anterior visualisation and ideal graft placement [1]. The advantages of this technique are its wide angle of visualisation and its applicability to all types of perforations [1, 21]. Disadvantages include anterior blunting, graft lateralisation, iatrogenic cholesteatoma, longer healing time due to more invasive procedure and longer operative time [1, 20–22]. The main advantage of the medial graft technique is that it is free from these disadvantages of the overlay technique [1, 20, 21]. In addition, it is faster and easier to apply and has a higher success rate compared to the overlay technique [1, 20, 21, 23]. The main disadvantages of this technique are the limited visual angle and the medialisation of the anterior part of the graft in subtotal and anterior perforations if adequate support is not provided [1, 20, 21]. TEES, with its wide visual angle, contributes positively to the difficulty of the anterior part of the medial technique.

In annular chondroperichondrial tympanoplasty, we used the under-over technique, a medial technique. The aim was to avoid the potential disadvantages of overlay tympanoplasty and to achieve a higher graft success rate. The type of graft material used is also important for the success of graft integration. The literature generally reports that cartilage and cartilage-perichondrium composite grafts have similar or higher success rates than temporalis fascia [1]. In their meta-analyses, Dr Yang et al. and Dr Mohamad et al. reported that cartilage grafts had better graft integration than fascia and similar results in hearing scores [14, 24]. Dr Varma et al. compared perichondrium and perichondrium-cartilage composite graft in their study and found that perichondrium-cartilage composite graft was more successful in graft integration [25]. Our technique also uses a perichondrium-cartilage composite graft. The difference to the composite grafts studied in the literature is that approximately 1 mm of cartilage is removed annularly from the peripheral edge of the graft and the peripheral 1 mm consists only of perichondrium. With this technique, we believe we have avoided the positional difficulties caused by cartilage thickness, especially in subtotal perforations, and contributed to the success of graft integration by placing the graft under the residual membrane. In the patients enrolled in the study, 95% of patients had successful graft integration and the patients with failure required minor revision.

Another advantage of our technique is its ease of application both microscopically and endoscopically. Although it can be performed by either method, in our personal practice we have often preferred endoscopic surgery in order to have the advantages of the endoscopic method. In the limited cases where endoscopic application was not possible, we could easily apply the technique using microscopic surgery. All patients included in this study underwent endoscopic surgery. While the temporal muscle fascia is often used in postauricular surgery, many researchers performing endoscopic surgery prefer the tragal chondroperichondrial graft [1, 8, 16]. The elasticity of the cartilage and its resistance to pressure changes, easy availability, availability within the surgical field and cosmetic acceptability play a role in this preference [1, 14]. In addition, the orientation and placement of cartilage grafts is more advantageous than fascia in endoscopic surgery, where surgery is performed with one hand [16]. However, there are authors who state that this advantage becomes a challenge in paediatric patients and patients with a narrow external auditory canal [20]. We believe that the annular chondroperichondrial graft pushes the boundaries of endoscopic use of cartilage grafts in this respect. With the circular cartilage that we have removed from the centre of the graft and the malleus slit that we have created, we believe that we have further facilitated the flexibility and single-handed management of our graft. The graft, which is now more pliable even in small or narrow external auditory canals, can be advanced to the target area as desired without being affected by possible physical difficulties.

The gain achieved with the circular cartilage removed from the centre of the graft is not only at the level of flexibility. The stiffness, resistance and mass of the cartilage are reduced accordingly, resulting in a graft material that is more suitable for hearing physiology. Studies have shown that acceptable hearing results can be achieved with cartilage tissue thinner than 0.5 mm [16, 26]. In the literature, the average tragus cartilage thickness was calculated to be 1.228 ± 0.204 mm in men and 1.090 ± 0.162 mm in women [26]. With our technique, in which the circular cartilage was removed from the centre of the graft, we utilised both the elasticity and compressive strength of the cartilage tissue and the low-resistance mass and stiffness of the perichondrium graft, which are better suited to hearing physiology, in a single graft. The mean pre-operative ABG of the patients in the study was 22.1, while the mean post-operative ABG was 5.7. The mean ABG gain was 16.4. All patients with a preoperative conductive hearing loss in the range of 10–50 dB regressed to the normal range of 0–20 dB postoperatively. Again, our technique overcame the difficulty of middle ear follow-up due to the semi-permeability of the cartilage. The perichondrium tissue in the centre allows the postoperative middle ear to be followed with its semi-permeable feature. With this feature, we believe that our technique will be a more ideal method, especially in patients with risk factors for cholesteatoma.

## Conclusion

In parallel with the development of otological surgical technologies, endoscopy is becoming increasingly important in tympanoplasty. We believe that our technique, endoscopic annular chondroperichondrial tympanoplasty, will be an important step in the search for the ideal graft material and technique.

**Table 3:** Patients’ pre- and post-operative air-bone-gap results

	Pre-operative	Post-operative
0–10	-	19
11–20	3	21
21–30	23	-
31–40	11	-
> 40	3	-
